# SURGICAL MANAGEMENT OF LARGE HEPATOCELLULAR CARCINOMA: THE FIRST SINGLE-CENTER STUDY FROM WESTERN INDIA

**DOI:** 10.1590/0102-672020190001e1505

**Published:** 2020-11-20

**Authors:** Prasad WAGLE, Rajvilas NARKHEDE, Gunjan DESAI, Prasad PANDE, D R KULKARNI, Paresh VARTY

**Affiliations:** 1 Lilavati Hospital and Research Centre, A-791, Bandra Reclamation, Bandra (West), Mumbai-400050, India; 2 Balabhai Nanavati Superspeciality Hospital, Mumbai, Maharashtra- 400056, India

**Keywords:** Hepatocellular carcinoma, Hepatectomy, Liver failure, Carcinoma hepatocelular, Hepatectomia, Insuficiência hepática

## Abstract

**Background:**

Majority of patients with large size HCC (>10 cm) are not offered surgery as per Barcelona Clinic Liver Cancer (BCLC) criteria and hence, their outcomes are not well studied, especially from India, owing to a lower incidence.

**Aim:**

To analyze outcomes of surgery for large HCCs.

**Methods:**

This retrospective observational study included all patients who underwent surgery for large HCC from January 2007 to December 2017. The entire perioperative and follow up data was collected and analyzed.

**Results:**

Nineteen patients were included. Ten were non-cirrhotic; 16 were BCLC grade A; one BCLC grade B; and two were BCLC C. Two cirrhotic and three non-cirrhotic underwent preoperative sequential trans-arterial chemoembolization and portal vein embolization. Right hepatectomy was the most commonly done procedure. The postoperative 30-day mortality rate was 5% (1/19). Wound infection and postoperative ascites was seen in seven patients each. Postoperative liver failure was seen in five. Two cirrhotic and two non-cirrhotic patients had postoperative bile leak. The hospital stay was 11.9±5.4 days (median 12 days). Vascular invasion was present in four cirrhotic and five non-cirrhotic patients. The median follow-up was 32 months. Five patients died in the follow-up period. Seven had recurrence and median recurrence free survival was 18 months. The cumulative recurrence free survival was 88% and 54%, whereas the cumulative overall survival was 94% and 73% at one and three years respectively. Both were better in non-cirrhotic; however, the difference was not statistically significant. The recurrence free survival was better in patients without vascular invasion and the difference was statistically significant (p=0.011).

**Conclusion:**

Large HCC is not a contraindication for surgery. Vascular invasion if present, adversely affects survival. Proper case selection can provide the most favorable survival with minimal morbidity.

## RESUMO

**Racional:**

A maioria dos pacientes com CHC de grande porte (>10 cm) não tem indicação cirúrgica conforme os critérios do Barcelona Clinic Liver Cancer (BCLC) e, portanto, seus resultados não são bem estudados, principalmente na Índia, devido a uma menor incidência.

**Objetivo:**

Analisar os resultados da cirurgia para HCCs de grande porte.

**Métodos:**

Este estudo observacional retrospectivo incluiu todos os pacientes submetidos à cirurgia para grandes CHC de janeiro de 2007 a dezembro de 2017. Todos os dados perioperatórios e de acompanhamento foram coletados e analisados.

**Resultados:**

Dezenove pacientes foram incluídos. Dez não eram cirróticos; 16 eram BCLC grau A; um BCLC grau B; e dois eram BCLC C. Dois cirróticos e três não-cirróticos foram submetidos à quimioembolização transarterial sequencial pré-operatória e embolização da veia porta. Hepatectomia direita foi o procedimento mais comumente realizado. A taxa de mortalidade pós-operatória em 30 dias foi de 5% (1/19). Infecção da ferida e ascite pós-operatória foram observadas em sete pacientes cada. Insuficiência hepática pós-operatória foi observada em cinco. Dois pacientes cirróticos e dois não cirróticos apresentaram vazamento de bile no pós-operatório. O tempo de internação foi de 11,9±5,4 dias (mediana de 12 dias). A invasão vascular estava presente em quatro pacientes cirróticos e cinco não cirróticos. O acompanhamento médio foi de 32 meses. Cinco pacientes morreram no período de acompanhamento. Sete tiveram recorrência e sobrevida mediana livre de recorrência foi de 18 meses. A sobrevida livre de recorrência cumulativa foi de 88% e 54%, enquanto a sobrevida global cumulativa foi de 94% e 73% em um e três anos, respectivamente. Ambos eram melhores em não-cirróticos; no entanto, a diferença não foi estatisticamente significante. A sobrevida livre de recidiva foi melhor nos pacientes sem invasão vascular e a diferença foi estatisticamente significante (p=0,011).

**Conclusão:**

CHC grande não é contraindicação para cirurgia. Invasão vascular, se presente, afeta adversamente a sobrevida. Seleção adequada de casos pode fornecer sobrevida mais favorável com morbidade mínima.

## INTRODUCTION

Hepatocellular carcinoma (HCC) is the fifth most common cancer worldwide^34^. Its incidence has tripled since 1980, especially in South Asia, Africa and North America^34^ ^,^ ^27^. However, the incidence in India is lower compared to the other south Asian countries^25^. Between 15% and 60% patients have non-specific symptoms like vague upper abdominal pain, anorexia, and weight loss and hence, have large tumors at diagnosis^34^ ^,^ ^5^. Surgical resection, ablation, and liver transplantation are currently the only curative therapies available for HCC, whereas trans-arterial liver-directed therapies and systemic chemotherapeutic agents are utilized in setting of advanced HCC either as downstaging or bridging therapy, or palliative treatment^15^.

Indian subcontinent has a predominance of patients with solitary and frequently large size HCC (>10 cm) at the time of diagnosis^34^ ^,^ ^25^ ^,^ ^15^. For the majority of these patients are not offered surgical resection, based on standard Barcelona Clinic Liver Cancer (BCLC) criteria. 

This article focuses on outcomes of surgical resection of these large HCCs at a single tertiary care center in Western India.

## METHODS

The study was performed after the approval of research protocols by the Ethics Committee of Lilavati Hospital and Research Centre, Mumbai, India, in accordance with international agreements (World Medical Association Declaration of Helsinki “Ethical Principles for Medical Research Involving Human Subjects,” amended in October 2013, www.wma.net). Written informed consent was obtained from the patient involved.

This retrospective observational study included all patients who underwent hepatic resection for large hepatocellular carcinoma (HCC) (tumor size ≥10 cm) from January 2007 to December 2017 in the department of gastrointestinal surgery at a tertiary care center^36^. Hospital database was screened to collect the preoperative work up, intraoperative details and postoperative as well as follow up data which was entered in a dedicated proforma. 

The preoperative data included demographic details, mode of presentation, pre-existing liver disease, degree of liver dysfunction, portal hypertension and preoperative blood investigations including complete blood counts, serum alpha fetus-protein and liver functions tests. All patients underwent a triphasic liver protocol computed tomogram (CT) and/or a magnetic resonance imaging (MRI) scan and high-resolution chest CT for diagnosis, staging, assessing operability and surgical planning. Radiological parameters including size and location of tumor, presence of satellite nodules, vascular invasion, tumor thrombus, presence of ascites, liver echotexture and nodularity, and signs of portal hypertension were noted. 

Liver biopsy was performed only in cases with diagnostic uncertainty on triphasic CT/MRI. The future liver remnant (FLR) volume was calculated preoperatively using automated Myrian 3-dimensional volumetry software (Intrasense, Montpellier, France) according to the plan of surgery. The cut off for remnant liver volume was 30% for normal liver and 40% for cirrhotic liver (Child A). Preoperative sequential trans-arterial chemoembolization (TACE) and portal vein embolization (PVE) was performed for a marginal remnant liver volume. 

The patients were preoperatively optimized. Our preoperative antibiotic prophylaxis included a third-generation cephalosporin 1 h prior to the incision and repeated four hourly intraoperatively. Antithrombotic prophylaxis was appropriately administered. After complete evaluation and preoperative optimization, hepatectomy was performed through a modified Makuuchi incision.

After careful examination and ruling out any extra hepatic disease, hilum was looped for Pringle maneuver. No other vascular exclusion procedures were preferred. Cavitron ultrasonic surgical aspirator, Kelly clysis, Habib 4X probe, Tissue link, and harmonic scalpel were the options utilized for parenchymal transection. Hemostasis and biliostasis was achieved with the help of titanium clips, sutures or bipolar coagulation. Selective (ipsilateral) inflow occlusion was performed routinely before parenchymal transection. Pedicle control was taken either with vascular stapler or sutures. A tube drain was placed in the right sub-hepatic region for all surgeries for large HCC. 

Histopathological data record included type of HCC, margin status, satellite nodules and microvascular and macrovascular invasion. Postoperatively, the patients were monitored in the surgical intensive care unit for minimum 48 h. The postoperative complications were diagnosed as per standard guidelines. Surgical site infection could be superficial incisional, deep incisional and organ space, which were diagnosed as per the CDC guidelines^6^.

Postoperative liver failure was defined per International Study Group for Liver Surgery as postoperatively acquired deterioration in the ability of the liver to maintain its synthetic, excretory, and detoxifying functions, which are characterized by an increased INR and concomitant hyperbilirubinemia on or after postoperative day 5a simple and easily applicable definition of posthepatectomy liver failure was developed by the International Study Group of Liver Surgery. Furthermore, a grading of severity is proposed based on the impact on patients’ clinical management. RESULTS No uniform definition of posthepatectomy liver failure has been established in the literature addressing hepatic surgery. Considering the normal postoperative course of serum bilirubin concentration and International Normalized Ratio, we propose defining posthepatectomy liver failure as the impaired ability of the liver to maintain its synthetic, excretory, and detoxifying functions, which are characterized by an increased international normalized ratio and concomitant hyperbilirubinemia (according to the normal limits of the local laboratory^30^. Bile leak was defined and graded by this international group as fluid with an increased bilirubin concentration in the abdominal drain or in the intra-abdominal fluid on or after postoperative day 3, or as the need for radiologic intervention because of biliary collections or relaparotomy resulting from bile peritonitis^14^.

Pneumonia was defined as new lung infiltrate plus clinical evidence that the infiltrate was of an infectious origin, which included the new onset fever, purulent sputum, leukocytosis, and/or a decline in oxygenation. Postoperative complications were recorded from the hospital records till the discharge and out-patient department follow up, and were graded according to Clavien-Dindo Classification^9^. The follow up protocol for all patients included the first follow up one month after surgery, then three monthly for two years, and thereafter six monthly. They were followed up with complete blood counts, liver function tests, alpha fetus-protein levels and abdominal ultrasound at each follow up and CECT for suspicious findings on ultrasound as well as yearly for the first two years. Follow up data also included duration of use of sorafenib if used, recurrence and its management, and mortality. 

### Statistical analysis

The data was meticulously entered in Microsoft Excel version 2016 and was analyzed with the help of SPSS version 20 software. The descriptive data was expressed in terms of mean±standard deviation, median and range. The nominal data and ordinal data was compared with the help of student t-test and Chi square test respectively. Survival data was analyzed using Kaplan-Meier Curve and was compared with life tables. P-value <0.05 was considered as significant for statistical association.

## RESULTS

A total of 19 patients underwent hepatectomy for large HCC and were included in this analysis. Table 1 shows the demographic profile of the patients and their preoperative assessment. Out of nineteen patients, 15 (79%) were males and four (21%) females. Ten patients were non cirrhotic. The etiology for cirrhosis was hepatitis B virus in four (44%), hepatitis C virus in three (33%), alcohol in one (11%) and non-alcoholic fatty liver disease in two (22%) patients. Mean age of the patients was 54 years (28-82), which was comparable in the cirrhotic and non-cirrhotic groups. Most common presentation was dull aching right and central upper abdominal pain seen in four (44%) cirrhotic patients and six (60%) non-cirrhotic. Seven (37%) were diagnosed incidentally whereas two patients, one from each group, presented with hemoperitoneum related to tumor rupture. Alpha fetus-protein was elevated in seven (78%) cirrhotic and six (60%) non-cirrhotic patients. 

**TABLE 1:** Demographic profile and pre-operative parameters recorded

Parameter	Cirrhotic	Non-cirrhotic	All patients	p
Age	57±10.4	51.5±16.1	54.1±13.6	0.39
Gender Male Female	09 07 02	10 08 02	19 15 04	0.91
Etiology for cirrhosis				
Hepatitis B infection	04	01	05	0.09
Hepatitis C infection	03	00	03	0.058
Alcohol Intake	01	00	01	0.28
Others	02	00	02	
Asymptomatic	04	03	07	0.78
Symptomatic Abdominal Pain Hemoperitoneum	05 04 01	07 06 01	12 10 02	
Elevated alpha-fetus-protein	07	06	13	0.41
Satellite nodules	01	00	01	0.28
BCLC grading A B C	09 6 1 2	10 10 0 0	19	0.28
Performance score 0 1	07 02	08 02	15 04	0.91
Trans-arterial embolization (TAE)	01	01	02	0.93
Sequential TACE- portal vein embolization	02	03	05	0.70
Biopsy	01	02	03	0.59

The preoperative diagnosis, based on imaging was established in 16 patients (84%). Percutaneous biopsy was done in the remaining three due to inconclusive imaging features. Sixteen were BCLC grade A, one cirrhotic was BCLC grade B, and two with vascular invasion evident on preoperative imaging were classified as BCLC C. The mean size of the tumor on CT was 12.9±2.3 cm (10-17) which was comparable in both the groups (13.3±2.1 cm in cirrhotic and 12.6±2.6 in non-cirrhotic; p=0.52). Satellite nodule was seen in one cirrhotic. 

Two patients presenting with tumor rupture and hemoperitoneum underwent trans-arterial bland embolization for initial bleeding control. Because of inadequate FLR, two from cirrhotic group, including one with tumor rupture and trans-arterial bland embolization and three from non-cirrhotic group underwent sequential TACE-PVE. The mean future liver remnant volume was 45.7±10.5% (30-70) which was comparable in both the groups. 

The details of surgical procedures performed are described in Table 2. Right hepatectomy was the most commonly done procedure. Pringle’s maneuver was used for ipsilateral inflow occlusion in 14 patients (six cirrhotic and eight non-cirrhotic). Cavitron ultrasonic surgical aspirator (n=11) was the most common technique utilized for parenchymal division followed by Kelly clysis (n=5). Median blood loss was 700 ml (mean 800±480 ml; range 200-2000 ml). Twelve patients required blood and/or blood products transfusion in perioperative period which was comparable in both the groups. One patient had intra-operative right hepatic vein injury resulting in significant blood loss which was managed by total vascular isolation of liver followed by primary repair. Intraoperative blood loss, requirement of blood products, type of technique used for parenchymal transection was comparable in cirrhotic and non-cirrhotic patients. 

**TABLE 2:** Intraoperative parameters observed in study groups

Parameters	Cirrhotics	Non-cirrhotics	All patients	p
Procedure Right hepatectomy (RH) RH with segment 4B excision Extended right hepatectomy Left hepatectomy Segment 5 & 6 excision	05 0 1 2 1	03 01 2 4 0	08 01 03 06 01	0.49
Tumor size (cm)	13.3±2.1	12.6±2.5	12.9±2.3	0.52
Remnant liver volume (%)	46.5±12.2	45.1±9.3	45.7±10.5	0.77
Blood loss (ml)	744±530	850±452	800±480	0.65
Intraoperative blood transfusions	05	07	12	0.53
Inflow occlusion	06	08	14	0.51
Total vascular exclusion	01	00	01	0.38
Technique CUSA (Cavitron Ultrasonic Surgical Aspirator) Habib 4X Harmonic scalpel Kelly clysis Tissue link	09 04 01 01 03 00	10 07 00 00 02 01	19 11 01 01 05 01	0.41

**TABLE 3:** Postoperative parameters recorded in study groups

Parameters	Cirrhotics	Non-cirrhotics	All patients	p
Intensive care unit stay (days)	2-7	2-8	2-8	0.85
Full mobilization (days)	3-5	3-5	3-5	0.96
Postoperative ventilation (days)	1	1-2	1-2	0.62
Postoperative liver failure A B	03 03 00	02 01 01	05 04 01	0.33
Bile leak	2	2	04	0.91
Ascites	2	5	07	0.28
Pleural effusion	01	01	02	0.94
Wound Infections	04	03	07	0.52
Clavein-Dindo (CD) grading of postoperative complications				
1/2/3/4/5	2/1/2/0/0	3/2/1/0/1	5/3/3/0/1	
CD grade ≥3	02	02	04	0.91
Recurrence	04	03	07	0.43
Histopathological examination Well differentiated HCC Moderately differentiated HCC Fibrolamellar HCC	08 01 00	05 00 05	13 01 05	0.036
Vascular invasion Microvascular invasion MacroVascular invasion	04/09 03 01	05/10 03 02	09 06 03	0.76
Hospital stay (days)	10.2±3.4	13.4±6.6	11.8±5.4	0.22
Recurrence	04	03	07	0.52
Mortality	01	00	01	0.25

The postoperative details and complications recorded are shown in Table 3. The mean stay in intensive care unit was two days. The postoperative 30-day mortality rate was 5% (1/19). This patient with intraoperative right hepatic vein bleed had postoperative liver failure secondary to outflow obstruction due to its thrombosis followed by cerebral vascular injury and expired 48 h after surgery. Wound infection and postoperative ascites was the most common morbidity seen in seven patients each. Postoperative liver failure was seen in five (grade A=4; grade B=1). Two patients in cirrhotic group and two in non-cirrhotic had postoperative bile leak which required intervention (percutaneous drainage in three and endoscopic retrograde cholangiopancreatography and stenting in one). The morbidity was comparable in both groups. The mean hospital stay for patients undergoing hepatectomy was 11.9±5.4 days (median 12 days) which was comparable in both groups. 

The final histopathology confirmed the diagnosis of HCC. Well differentiated HCC was seen in 13 (eight cirrhotic and five non-cirrhotic), moderately differentiated in one cirrhotic whereas fibrolamellar variant was seen in five non-cirrhotic. Vascular invasion was present in present in four cirrhotic and five non-cirrhotic patients (p=0.81). Macrovascular invasion was seen in one cirrhotic and two non-cirrhotic. Surgical margins were negative in all. 

The median follow-up was 32 months (8-100). Two patients were lost to follow up after 35 and 84 months respectively. The 30-day postoperative mortality was 5%. Five died in the follow-up period of which one died due to sequelae of chronic liver disease at 60 months without any evidence of disease recurrence and four due to recurrent and/or metastatic disease. Seven patients (36%) had recurrence in the follow-up period with range of 9-30 months and median recurrence free survival (RFS) was 18 months. One patient underwent surgical resection for operative bed recurrence followed by sorafenib therapy for a period of three years and is currently on follow up. One patient underwent TACE for a local recurrence whereas three were treated with sorafenib who have controlled pulmonary metastasis on therapy. Two patients refused further treatment. 

**TABLE 4:** Recurrence free survival (RFS) and overall survival (OS) observed

Recurrence free survival (RFS)	Cirrhotics (n=9)	Non-cirrhotics (n=10)	All patients (n=19)	p
At 1 year (%)	75	100	88	0.405
At 3 years (%)	45	62	54	
Overall survival (OS)				
At 1 year (%)	89	100	94	0.199
At 3 years (%)	76	72	73	
At 5 years (%)	0	72	48	

**FIGURE 1:**
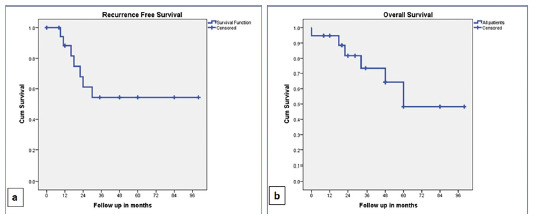
Kaplan-Meier curves: A) recurrence free survival (RFS); B) overall survival (OS) in all patients

**FIGURE 2:**
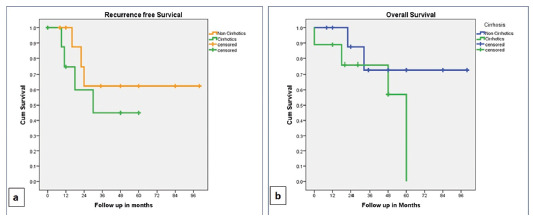
Kaplan-Meier curves: A) recurrence free survival (RFS); B) overall survival (OS) in cirrhotic and non-cirrhotic

**FIGURE 3:**
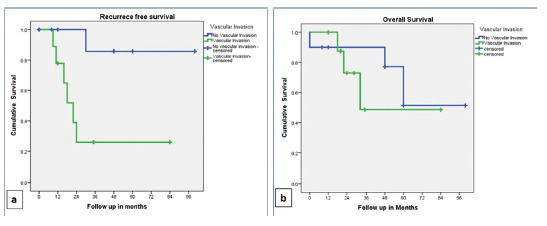
Kaplan-Meier curves: A) recurrence free survival (RFS); B) overall survival (OS) in patients with and without vascular invasion

The cumulative RFS was 88% and 54% at one year and three years respectively, whereas the cumulative overall survival (OS) was 94% and 73% at one and three years respectively. The RFS and OS were better in non-cirrhotic as compared to cirrhotic; however, the difference was not statistically significant. The survival data is elaborated in Table 4 and Figures 1 and 2. The RFS was better in patients without vascular invasion compared to those with it as shown in Figure 3, and the difference was statistically significant (p=0.011).

## DISCUSSION

HCC is very common in south Asia and Africa and is showing a rise in incidence in North America^34^ ^,^ ^27^. In a study from India, only 10.7% of 324 patients could be offered hepatic resection for HCC^26^. Hence, there are very few studies from India on outcomes of surgical resection of HCC and the numbers for large HCC are even smaller. Ours is the first single center series on outcomes of surgical management of large HCC from Western India. 

HCC is predominantly seen in males across studies as in our study with male: female ratio of 15:4. The age distribution of HCC shows two peaks in an Indian study, one at 40-55 years and the other above 60 years^5^. The median age here is 54 years. Liver cirrhosis, one of the most common risk factor for development of HCC, was present in 47.36% of our patients comparable to study by Liu et al and Chen et al.^21^ ^,^ ^7^ HBV infection was most common etiology for cirrhosis present in 44% followed by HCV infection (33%) in our study. In others patients with large HCC, the cause for cirrhosis was HBV and HCV infection in 43%-77% and 4.3%-29% respectively. Most tumors (63%) were symptomatic on presentation owing to its large size which is comparable to existing literature^25^.

Historically, large tumors have been considered to have a poor prognosis due to its association with vascular invasion, satellite nodules and distant intrahepatic or extrahepatic metastasis^18^. Major hepatic resection is a complex surgery with significant morbidity and mortality and hence, appropriate case selection is very important. With growing expertise, technology, and understanding of hepatic physiology and intraoperative anesthetic requirements, the surgical risks have reduced over years when performed at high volume centers by experienced surgeons. A lot of centers are now performing liver resections for selected patients with large HCC and the outcomes are improving with extending indications^8^.

The management options for large HCC include resection, liver transplantation, TACE/trans-arterial radioembolization (TARE), cytoreductive strategies like hepatic artery ligation, radiofrequency/microwave ablation or infusion chemotherapy and sorafenib. Sequential TACE followed by PVE is also utilized as a strategy to augment future liver remnant as discussed later^12^ ^,^ ^29^. Out of these options, the curative options are resection and transplantation which would be beneficial only if the survival after these procedures is better than after TACE/TARE or sorafenib^18^ ^,8,^ ^35^. To stratify the risk-benefit as per these management options, various staging systems have been utilized for HCC. These have evolved over the years and currently the BCLC staging system and the Hong Kong staging system are the two commonly used systems to plan treatment^12^. 

As per BCLC, which is also routinely followed in India, large HCC is deemed resectable only for a patient who has no extrahepatic disease or vascular invasion, no portal hypertension, Child-Pugh class A and a preserved performance status. For all the patients not meeting this criteria, standard non-surgical treatment provide a median survival of 41 months for TACE and 12 months for sorafenib as per BCLC staging^18^. In our study, two BCLC C patients and one BCLC B have also been selected for surgery.

In Asia, TACE is used in small tumors with intermediate liver function precluding surgery^35^. In a randomized controlled trial, the mean size of tumors treated by chemo-embolization was 5.2 cm^23^. Is described that 44% had a mean diameter >5 cm. Despite tumor size, a beneﬁt in terms of survival in selected patients (preserved liver function, performance status <2, and Okuda stage II) was observed after TACE, compared with a control group receiving no treatment^23^. Tumor size >5 cm was historically considered a negative predictive factor affecting overall survival after chemoembolization^22^. A combination of TACE and radiofrequency or high intensity focused ultrasonography has demonstrated better results compared with chemoembolization alone, both in terms of tumor response and overall survival^40^ ^,^ ^41^. However, recent studies suggest that surgical resection leads to better survivals than TACE, either alone or in combination with radio-frequency ablation^21^.

Liver transplantation for large HCC >10 cm is essentially not a treatment option as per the standard Milan or University of California, San Francisco criteria^24^ ^,^ ^44^. With the advent of living donor transplantation, indications are being extended to these patients. However, they have a lower survival benefit because of a higher recurrence rate after transplantation. It has also been used for salvage treatment for recurrence after resection for a large HCC. Few studies exclude the patients with giant HCC and those with vascular invasion to optimize the survivals of the recipient after liver transplantation and it is not a primary treatment option for large HCC >10 cm^38^. None of our patients underwent liver transplantation.

Hence, the only curative treatment available to provide the greatest survival benefit in this group of patients is surgical resection^18^. Recent studies have aimed at identifying factors to predict a poor prognosis after surgery and thereby not consider these patients for a complex and challenging hepatic resection^12^ ^,^ ^23^ ^,^ ^38^. A study identified elevated bilirubin levels (>5.8 mg/l), platelet count <1.5 l and portal vein tumor thrombus as independent predictors of three months mortality and portal vein tumor thrombus as the sole risk factor for early recurrence related mortality. Size was not identified as a risk factor in this study^18^. Abdalla et al^1^ found that tumor size had a direct correlation with vascular invasion and a significantly higher proportion of patients with tumors >5 cm in size had vascular invasion. However, tumor size alone fails to correlate with survival, and in patients with a single tumor, only vascular invasion signiﬁcantly affects the prognosis, irrespective of tumor size^1^ ^,^ ^18^ ^,^ ^28^. 

Multicentricity has been found in patients with large HCC and this in presence of cirrhosis is considered a part of the cirrhotic pathology rather than inoperable disease^39^. Combination of TACE/TARE, radiofrequency with surgery can also be utilized in these cases where surgery alone is deemed inadequate. This is true even for bilobar disease in presence of cirrhosis^38^. Only one of our patients had a satellite nodule and none had multicentric disease. Once the patient is found to have resectable disease, the next and the most important assessment is for future liver remnant which is done using CT volumetry at our center. 

Future liver remnant is considered adequate when it is more than 20% (normal liver), 30% (metabolic syndrome, steatohepatitis) and 40% (cirrhotic liver) of the total liver volume. In cases with inadequate future liver remnant, the options are PVE/associating liver partition and portal vein ligation for staged hepatectomy to facilitate the growth of future liver remnant or TACE/TARE to downsize the tumor or a combination of these procedures^19^ ^,^ ^32^. To prevent tumor progression during the waiting period after PVE, TACE followed by PVE is being preferred for large HCC in patients with hepatic fibrosis, cirrhosis, steatosis or steatohepatitis^3^ ^,^ ^31^. Recently, biembolization has been suggested wherein PVE is combined with one or two hepatic vein embolization to achieve complete liver venous deprivation. This approach, however, needs more studies^11^. We prefer sequential TACE-PVE in our cases with inadequate future liver remnant.

Minimally invasive approaches are now being utilized for liver resection. Recent studies have shown that large HCC cannot be viewed as a contraindication to laparoscopic liver resection provided the surgical team is experienced to handle such cases^37^. Southampton consensus guidelines for laparoscopic liver resection also consider laparoscopy as a feasible option for large HCC^2^. A study has shown greater blood loss, higher conversion rates (18.4%), more frequent and prolonged pedicle clamping times, longer operating times and a longer hospital stay for laparoscopic liver resection. However there was no difference in morbidity, mortality and long-term outcomes^16^. In another study, it showed higher blood loss and longer operative times for tumors >10 cm compared to tumors of 5-10 cm size without significant differences in the morbidity and mortality rates^33^. Survival is non-inferior compared to open resection across studies^13^. 

A few special considerations are worth mentioning pertaining to liver resection for large HCC. Owing to a large tumor, approach may vary from a traditional subcostal or Makuuchi incision to a thoraco-abdominal approach to achieve early control of the supra-diaphragmatic vena cava^38^. Since the maneuverability of the tumor is difficult owing to its size and location, a hanging maneuver or an anterior approach may be required for large right sided tumors which has been shown to reduce blood loss due to decrease in the risk of venous avulsion during rotation of liver and reduced risk of tumor rupture. It also reduces cardiovascular and hepatic vascular abnormalities during surgery by limiting the torsion of inflow and outflow pedicles that may occur during liver rotation^20^. The inflow and outflow control need to be considered and kept ready before starting parenchymal division, and in case of major bleeding, total vascular exclusion may also be required^38^. We perform selective ipsilateral inflow occlusion for hepatic resections for large HCC. 

A retrospective study of 481 consecutive hepatic resections for HCC at a single center in China revealed 260 patients with solitary large HCC >5 cm in size^43^. Multivariate analysis revealed longer operating time, higher blood loss and transfusion requirement, and higher rate of postoperative infectious complications in these patients, compared to those with smaller tumors. However, the OS and disease free survival were not significantly different in the two groups^43^. A prospective study on 103 patients of HCC in 2017 revealed a higher perioperative mortality rate in patients with tumors larger than 5 cm, as compared to those with smaller tumors^41^. On univariate analysis, there was no statistically significant difference in disease free survival and OS amongst these two groups. In multivariate analysis, presence of lymphovascular emboli had a significant effect on the OS. In our study, total nine patients had vascular invasion out of which six had microinvasion and three had macroscopic invasion. 

Similarly, a retrospective study on 81 patients of HCC contained 75% patients with tumors >10 cm in size. In multivariate analysis, tumor size did not show any significant impact on the RFS and OS as can also be seen in our study^25^. However, the difference in RFS between patients with and without vascular invasion was significant. While those with tumors >10 cm were predominantly non-cirrhotic (78%), postoperative liver failure and ascites were more common in cirrhotic with large HCC^25^.

Large HCCs do have early recurrences, between 12-18 months after surgery ^25^ ^,^ ^10^ ^,^ ^42^. Panwar et al^25^ showed 55% recurrences, with a median RFS of 12 months, and 3-year RFS of 40%. Our study has an RFS of 88% and 54% at one and three years follow up, respectively. This is a bit higher than Fan et al^10^, which showed 38% 3-year RFS. Despite this, the OS across most studies has been good, reaching 60% to 80%^10^ ^,^ ^42^. This may be attributed to more aggressive treatment of the recurrences, including repeat hepatic resections, liver transplantation, ablation and TACE^4^ ^,^ ^42^.

Chen et al^7^ evaluated the outcomes of liver resection for 16 elderly patients with large HCC >10 cm and found 1, 2 and 3-year OS of 93.7%, 56.3% and 12.4%, respectively. Liau et al^17^ showed OS was 33% for both study groups with tumor <10 cm and >10 cm. Our study also shows a 94%, 73% and 48% overall survival at 1, 3 and 5 years follow up, respectively. Thus, surgery definitely provides better outcomes in well selected cases of large HCC. Patients with single large HCC and no vascular invasion have the most favorable outcomes after hepatic resection when performed by experienced surgeons at high volume centers.

## CONCLUSION

Large HCC by itself is not a contraindication for surgery. Vascular invasion is the only significant prognostic factor which adversely affects survival. Proper case selection, especially single tumor with no gross vascular invasion in a patient with good performance status, strategies for future liver remnant augmentation by sequential TACE-PVE, good preoperative planning, and adherence to principles of hepatic surgery can provide the most favorable survival with minimal morbidity. 
